# Initial clinical use of the intraocular endoscope holding robot in pars plana vitrectomy

**DOI:** 10.1007/s10384-025-01285-1

**Published:** 2025-10-03

**Authors:** Kohei Kiyohara, Keijiro Ishikawa, Kodai Yuge, Satoshi Yamana, Shintaro Nakao, Koh-Hei Sonoda

**Affiliations:** 1 Department of Ophthalmology, Graduate School of Medical Sciences, Kyushu University, Fukuoka, 812-8582 Japan; 2 Department of Ophthalmology, Graduate School of Medical Sciences, Juntendo University, Tokyo, Japan

**Keywords:** Robot, Intraocular endoscope, Vitrectomy, Assistant, OQrimo^®^

## Abstract

**Purpose:**

OQrimo^®^ is a robotic assistant system that supports vitreoretinal surgery by holding an intraocular endoscope or illumination device to assist the surgeon. In April 2023, Japan approved this system, and as the world's first clinical institution to implement OQrimo^®^, we aimed to evaluate its safety and clinical utility.

**Research design:**

Retrospective case series

**Methods:**

The study analyzed all vitreoretinal surgery utilizing OQrimo^®^ at Kyushu University Hospital between December 1, 2023 and November 31, 2024. Data collection included patient demographics, preoperative diagnoses, surgical procedures, equipment used, surgical records, and perioperative complications. We analyzed OQrimo's safety profile and patterns of clinical use based on these data.

**Results:**

Eight eyes from eight patients were included. Preoperative diagnoses included proliferative vitreoretinopathy, panuveitis, acute retinal necrosis, macular hole, and secondary glaucoma due to uveitis. The purpose of pars plana vitrectomy included silicone oil removal, vitreous biopsy, internal limiting membrane peeling, and Ahmed valve implantation via pars plana. OQrimo^®^ maintained stable endoscope positioning in all cases, enabling observation of the peripheral retina without scleral indentation. In seven cases, OQrimo's endoscopic visualization and a wide-viewing system were used simultaneously. No intraoperative or postoperative complications were observed in any case.

**Conclusion:**

We confirmed the safety of OQrimo^®^ during its initial clinical application. The system facilitated the observation of the peripheral retina using an intraocular endoscope without scleral indentation.

## Introduction

**Fig. 1:**
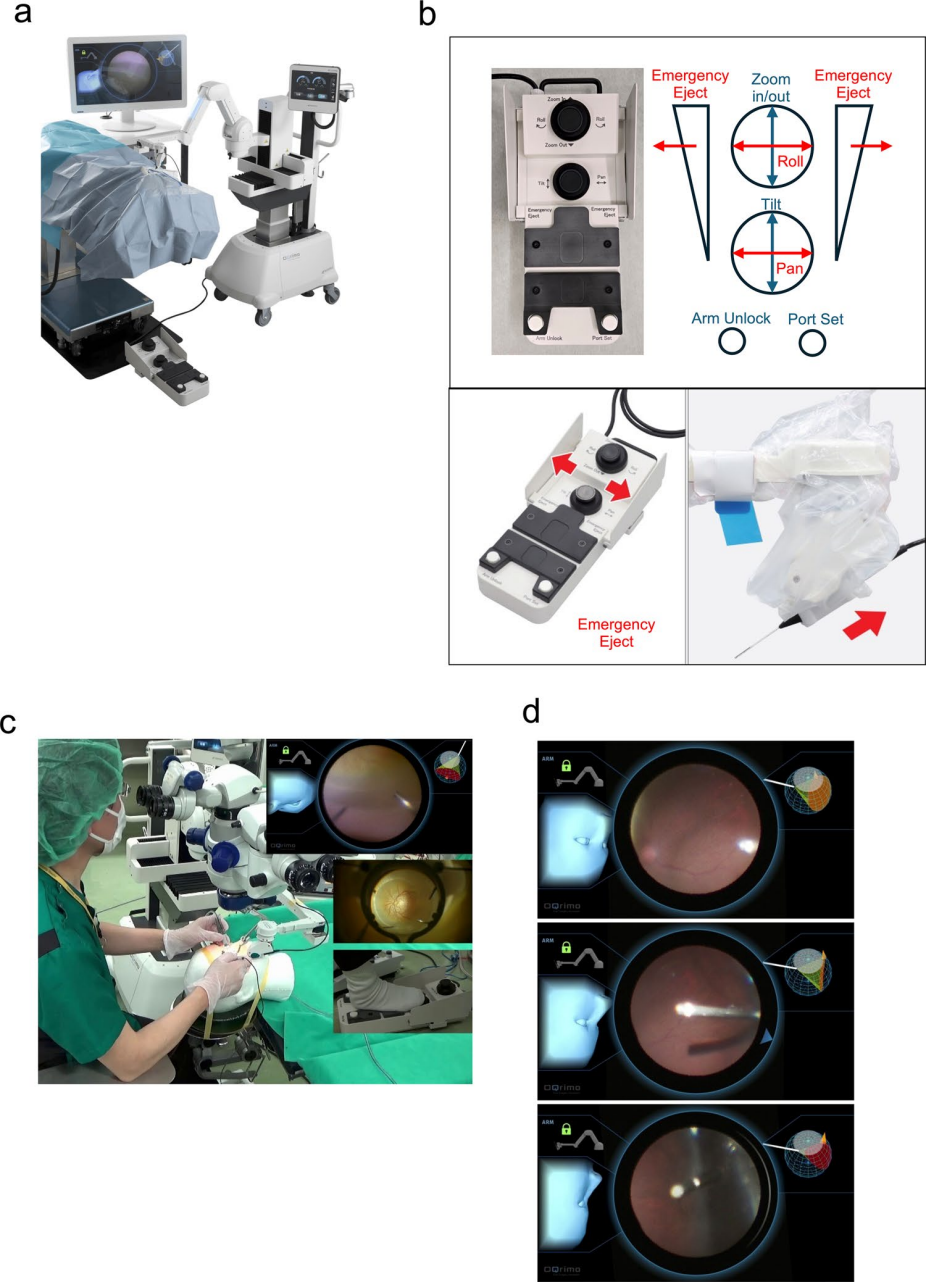
Intraocular endoscope-holding robot OQrimo^®^.** a** The total system features of OQrimo^®^ that stably hold an intraocular endoscope or illumination device. The surgeon can operate OQrimo^®^ using a foot controller while viewing the endoscope screen. **b** Foot controller with pan, tilt, zoom in, zoom out, roll, arm unlock, port set, and emergency eject functions (upper panel). When the operator senses danger, they can kick either the left or right wall of the foot controller (lower left panel) , allowing OQrimo^®^ to perform an emergency retraction of the instrument inserted into the eye (lower right panel). The reaction time for this process is 0.035 seconds. **c** OQrimo^®^ is designed for use in combination with a wide-viewing fundus observation system such as RESIGHT^®^. Upper left: View of the instruments at hand. Each tool can be inserted without interference. Upper right: OQrimo^®^ main display. Endoscopic images are projected. Middle right: Wide-angle fundus viewed with RESIGHT^®^. Lower right: Foot controller. **d** By continuously moving OQrimo^®^, it is possible to observe the contralateral pars plana and the posterior surface of the iris (upper→center→lower panels). The endoscopic image appeared in the center of the monitor. The monitor also features two side displays, allowing for a simulated “virtual eye mapping” function. The left display shows a head image viewed from OQrimo^®^, while the right display shows the “virtual area” illuminated by the OQrimo^®^ -holding endoscope (or illumination) in the eye. The orange indicates areas in the back of OQrimo^®^ insertion, while the red indicates areas in front of it.

Robotic-assisted surgery has rapidly gained widespread adoption in general surgery, with over one million procedures performed annually [1]. In ophthalmic surgery, various robotic systems have been developed, ranging from handheld stabilization devices to remote-controlled consoles [2–4]. However, the adoption of robotic systems in ophthalmic surgery remains limited. The challenges that hinder adoption are the extremely confined surgical field [5] and the need for precise motion control during intraocular manipulation [6]. To overcome these challenges and introduce robotics to ophthalmic surgery, we created the vitreoretinal surgery assistance robot OQrimo^®^ (Product name: Endoscope holder OQrimo^®^, Registration number: 13B1X10216000003) (Fig 1a).

OQrimo^®^ is a surgical assistance robot that provides surgeons with a third hand. The system features a robotic arm with a gimbal mechanism that stably holds an intraocular endoscope or illumination device. Gimbal mechanisms are also employed in other medical devices requiring high-precision position control, such as image stabilization systems and stereotactic radiotherapy [7]. This functional design concept represents an innovative approach to ophthalmic surgical robotics.

OQrimo^®^ offers two primary operating modes. In the endoscope holding mode, it enables observation of the peripheral retina without scleral indentation. In the illumination holding mode, it serves as a chandelier illumination system, allowing surgeons to manipulate with both hands. The system features an automatic emergency evacuation system that activates upon impact detection for added safety (Fig. 1 b).

The development of OQrimo^®^ began in 2012 as an industry-academia collaboration project involving Kyushu University, Tokyo Institute of Technology, Juntendo University, Yamaguchi University, and River Field Inc. [8]. The development was accelerated by research grants from AMED (Japan Agency for Medical Research and Development) in 2015 and 2017, in consultation with Japan's Pharmaceuticals and Medical Devices Agency (PMDA). The device received approval as a general medical device in April 2023, with the world's first clinical use initiated at Kyushu University Hospital in December of the same year. This study aimed to evaluate the safety and efficacy of OQrimo^®^ in initial clinical cases.

## Methods

### Research Design and Population

This single-center retrospective case series included all patients who underwent vitreoretinal surgery using OQrimo^®^ ‘s endoscope mode at the Department of Ophthalmology, Kyushu University Hospital, between December 2023 and November 2024. Data collection encompassed patient demographics (age, sex), clinical information (medical history, diagnosis, surgical procedure), and surgical records (equipment used, surgical videos, and endoscopic images).

### Regulatory and ethical considerations

OQrimo^®^ is approved as a Class I medical device in Japan. Class I devices are defined as having minimal risk to human health, even in the event of a malfunction. We initially consulted our hospital's Medical Safety Management Division and received valuable advice. As the world's first observation-based ophthalmic surgical assistant robot, and due to the significant need for attention and accountability, the Advanced Novel Medical Care Evaluation Subcommittee, following deliberations, approved it for routine clinical use. This study was approved by the Institutional Review Board of Kyushu University and conducted following the rules set by the Declaration of Helsinki. The requirement for informed consent was waived by the review board. We did not receive funding from any company and do not fall under the category of specific clinical research in Japan.

### Equipment

**Fig. 2:**
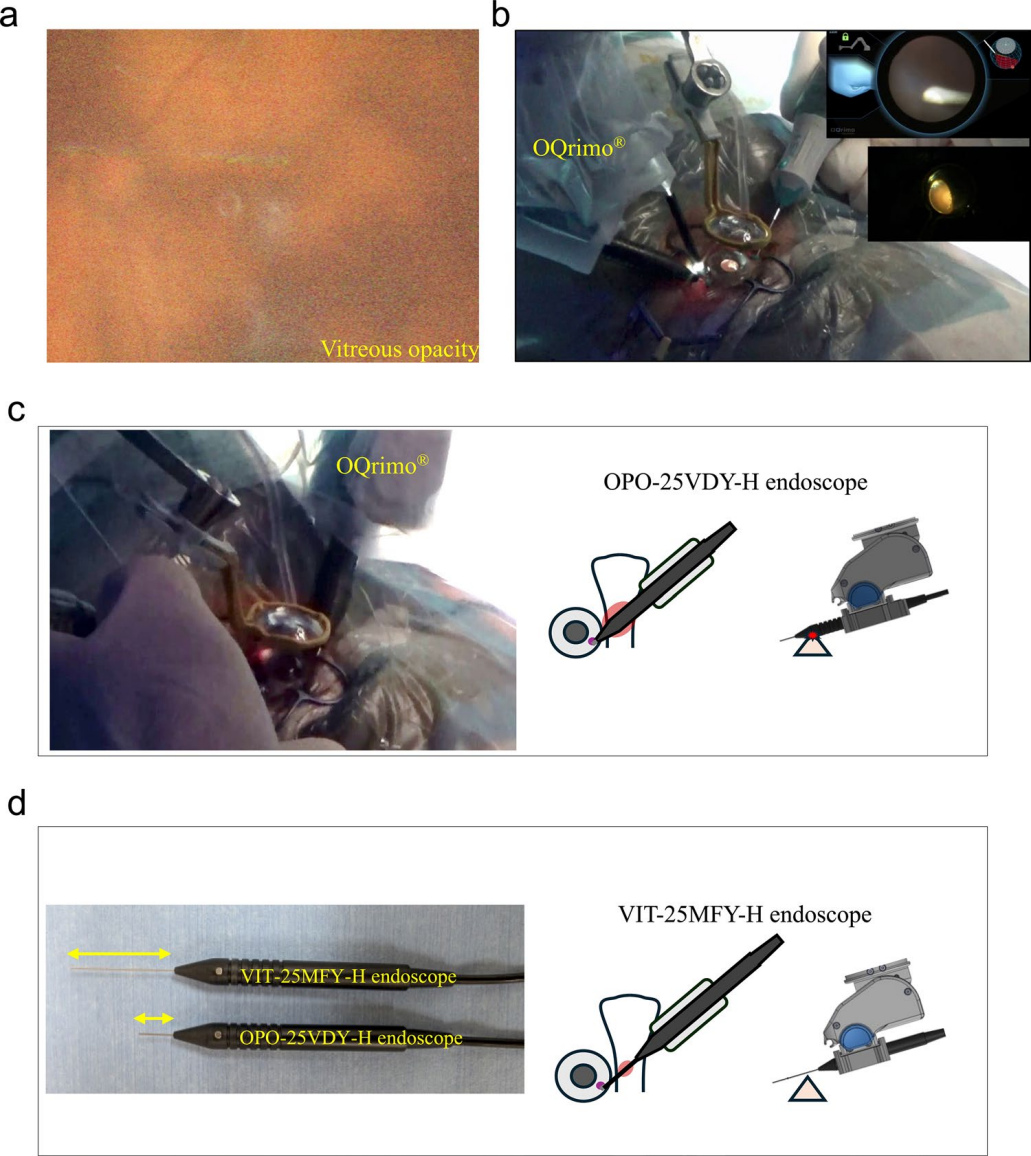
Use of OQrimo^®^ in Case 4.** a** Preoperative wide-angle fundus photograph of the left eye. Diffuse vitreous opacity is seen. **b** Resight and OQrimo^®^ being used together, with OQrimo^®^ holding the endoscope and the cutter in the right hand. The instruments can be manipulated without interference. The upper right of the screen shows the endoscope image, and the center right of the screen shows the microscope image. **c** The left panel shows the interference between patient’s nose and the body of the endoscope. The right panel shows the positional relationship between the face and the endoscope when interference occurs. **d** The left panel shows the feature of two different endoscopes. As shown in the right panel, the long insertion endoscope makes it easier to avoid interference (the right panel).

We used RESIGHT^®^ wide-viewing system (Carl Zeiss AG) during vitrectomy. We used intraocular endoscope systems manufactured by Machida Co., Ltd. in conjunction with OQrimo^®^ (Table 1a, b, Fig. 2d). Two types of endoscopes were employed: VIT-25MFY-H (a conventional model with a standard shaft length) and OPO-25VDY-H (a short-shaft model featuring an extended working distance and wider field of view designed to reduce the risk of over-insertion). Two endoscopic camera systems were used: MVH-2010A (a standard camera system) and MVH-3010R (a high-sensitivity camera system).

**Table 1:** Intraocular endoscope and camera systems manufactured by Machida Co., Ltd. (Abiko, Japan) in conjunction with OQrimo^®^.

a. Technical Specifications of OQrimo Endoscope Systems				
Model	Gauge size	Insertion length	Focal length	Medical device registration no.
VIT-25MFY-H	25 G	30 mm	5 mm	225AFBZX00002000
OPO-25VDY-H	25 G	10 mm	10 mm	304AFBZX00088000

b. Technical Specifications of Endoscopic Camera Systems					
Model	Image sensor type	Effective resolution	Sensitivity	Medical device registration no.	Key features
MVH-2010A	1/3-inch CMOS Sensor	1920 x 1080 pixels	F14 (2000 lux)	12B1X10015000041	Superior color reproduction
MVH-3010R	1/2.8-inch CMOS Sensor	1920 x 1080 pixels	F5.6 (2000 lux)	12B1X10015000046	Enhanced light sensitivity

### Surgeons

All cases in this series were consecutively operated on by the same surgeon—the only one who used OQrimo^®^ during the study period—who has more than 25 years of vitreoretinal surgery experience and has performed over 2,000 vitrectomy procedures.

## Results

The study included eight eyes from eight patients (four men and four women). The average age of the patients was 54.8 ± 21.7 years (95% confidence interval: 36.7-72.9 years). Table 2 presents detailed characteristics of the patients. All surgery was conducted by a single surgeon (KS). The preoperative diagnoses consisted of proliferative vitreoretinopathy (PVR) in three cases, panuveitis in two cases, acute retinal necrosis in one case, a macular hole in one case, and secondary glaucoma in one case. Aside from pars plana vitrectomy (PPV), the surgical procedures included silicone oil removal in four cases, vitreous biopsy in two cases, internal limiting membrane peeling in one case, and Ahmed valve implantation via pars plana in one case. For the endoscopic procedures, the OPO-25VDY-H endoscope with the MVH-3010R camera system was utilized in Case 4, while the VIT-25MFY-H endoscope with the MVH-2010A camera system was employed in all other cases.

**Table 2:** Detailed characteristics of the patients.

Patients’ characteristics, surgical methods and results												
Case	Age	Sex	Eye	VA (log MAR)	IOP (mmHg)	Lens stasus	Primary disease and indication for PPV	Anesthesia	Purpose of PPV	Intraoperative complication	follow up period	final status
1	72	M	OS	0.82	10	IOL	Expulsive hemorrhage / PVR	LA	SO removal	–	5M	Uneventful
2	67	F	OR	0.70	24	IOL	Sarcoidosis / PVR	LA	SO removal	–	12M	Good control of uveitis
3	46	M	OS	1	9	IOL	Acute Retinal Necrosis	LA	Silicone oil removal	–	7M	Uneventful
4	74	F	OS	1	12	IOL	Panuveitis / VO	LA	Vitreous biopsy	–	6M	Exclude ML、good control of uveitis
5	70	M	OS	0.097	12	phakia	Macular Hole	LA	ILM peeling and 20%SF6 gas injection	–	2M	Macular hole was closed
6	25	M	OS	1	11	IOL	Sarcoidosis / PVR	LA	SO removal	–	5M	Reoperation 4M later
7	69	F	OS	0.52	17	phakia	Panuveitis / VO	LA	Vitreous biopsy	–	4M	Diagnosed as Sarcoidosis, good control of uveitis
8	15	F	OR	0.079	27	IOL	JIA / Secondary Glaucoma	GA	Ahmed valve implantation via pars plana	–	3M	Good control of IOP (6 to 9 mmHg)

*M* Male, *F* Female, *OS* left eye, *OR* right eye, *VA* visual acuity, *IOP* intraocular pressure, *IOL* intraocular lens, *PPV* pars plana vitrectomy, *PVR* proliferative vitreoretinopathy, *VO* vitreous opacity, *JIA* juvnile idiopathic arthritis, *LA* local anesthesia, *GA* general anesthesia, *SO* silicone oil, *ILM* internal limiting membrane, *SF6* sulfur hexafluoride 6, *M* months

The RESIGHT^®^ wide-viewing system was used in all cases except Case 1, without any noted difficulties with OQrimo^®^. No case in this series employed a 3D heads-up visualisation system. In all cases the peripheral retina could be visualized through the endoscope, and in some instances, it was also possible to observe the ciliary body, posterior iris surface, and intraocular lens (Fig. 1c, d). The surgeon could operate OQrimo^®^ seamlessly using the foot controller (Fig. 1b). None of the cases required the surgeon to activate the emergency evacuation system. There were no intraoperative or postoperative complications in any of the cases. Here, we discuss cases 4, 6, and 8, which are presented below as representative examples.

### Case 4

The patient was a 74-year-old woman. She had vitreous opacity and anterior chamber inflammation OS, which did not improve with betamethasone eye drops three times a day and prednisolone 20 mg/day, so she was referred to our clinic for an initial examination.

During the initial examination, the left corrected visual acuity was 20/50, intraocular pressure was 9 mmHg, and the fundus appeared hazy with a large number of inflammatory cells in the anterior chamber, along with flare, hypopyon, and diffuse vitreous opacity (Fig. 2a). The patient was diagnosed with panuveitis OS. Despite increasing the betamethasone eye drops to six times a day and administering Sub-Tenon's Triamcinolone Acetonide injection, the vitreous opacity did not improve. Consequently, the decision was made to perform a vitrectomy for both diagnosis and treatment.

The patient underwent surgery with local anesthesia. To rule out intraocular malignant lymphoma, vitreous biopsy and pars plana vitrectomy were performed using OQrimo^®^. The preoperative visual acuity OS was 20/200, and the IOP was 12 mmHg. Surgery was performed as follows: four 25G ports were established, vitreous biopsies were performed, and the vitreous was then excised to the periphery.

We used OQrimo^®^ to examine the complications in the peripheral retina. The irrigation port was switched from the inferior temporal side to the inferior nasal side, and the endoscope was held by the OQrimo^®^ and inserted through the inferior temporal port, operated via a foot controller while monitoring the contralateral pars plana. The wide fundus image from the RESIGHT® system and the endoscope image could be viewed simultaneously (Fig. 2b). Next, the endoscope was inserted through the upper nasal port, facilitating observation of the contralateral side in the same manner. When the OQrimo^®^ was adjusted to observe the ciliary body, the patient's nose interfered with the endoscope (Fig. 2c). In this case, we had used the OPO-25VDY-H endoscope, which has a 10mm tip shaft, for the first time. When we switched to the original VIT-25MFY-H endoscope (with a 30mm tip shaft), the interference was resolved (Fig. 2d). OQrimo^®^ maintained stable endoscope positioning, enabling observation of the peripheral retina without scleral indentation. No complications or mechanical issues arose intraoperatively or postoperatively.

The biopsy results indicated no malignancy, effectively ruling out malignant lymphoma. The postoperative course was uneventful, with no recurrence of vitreous opacity observed one month after surgery.

### Case 6

**Fig. 3:**
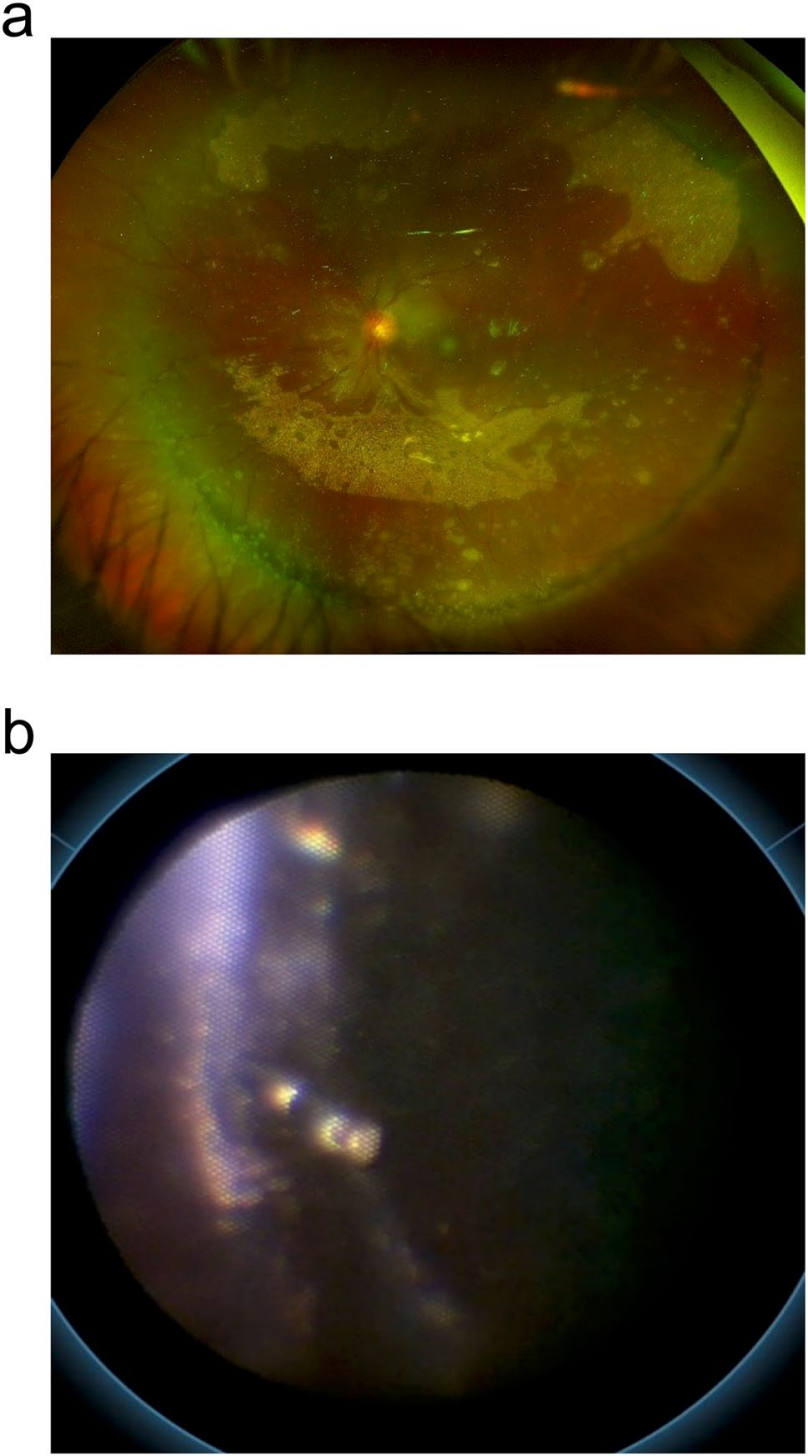
Use of OQrimo^®^ in Case 6.** a** Preoperative wide-angle fundus photograph of the left eye. The silicone oil is partially emulsified, and stick to entire retina. **b** The endoscopic view during the procedure shows residual silicone oil being removed by aspiration with a vitreous cutter, while an endoscope held by OQrimo® confirms the process.

The patient was a 25-year-old man referred to our hospital for bilateral granulomatous panuveitis and resistance to topical steroids and oral prednisolone. At our hospital, pulmonary sarcoidosis was suspected, a skin biopsy was performed, and it was subsequently confirmed as sarcoidosis. Examination of the left eye revealed pigmented keratic precipitates, anterior intravitreal cells, flare, posterior synechia, vitreous opacity, and a proliferative membrane covering the entire retinal surface. The patient underwent initial surgery for vitreous opacity OS, including PEA + IOL implantation and pars plana vitrectomy. However, retinal detachment was observed on the 8th postoperative day, prompting PPV, encircling, and silicone oil injection on the same day. Four months later, silicone oil extraction was performed using OQrimo^®^. Preoperative fundus photographs are shown (Fig. 3a).

Surgery was performed under local anesthesia; four 25G ports were created, and silicone oil was removed. Then, the irrigation port was moved from the inferior temporal side to the inferior nasal side, with the endoscope held by OQrimo^®^ and inserted through the inferior temporal port. By utilizing the RESIGHT^®^ wide-viewing system, both the wide-angle fundus image and the endoscope image could be viewed simultaneously. Subsequently, the endoscope was reinserted through the upper nasal port to observe the contralateral side. Residual emulsified silicone oil was found in the pars plana, which was then removed by aspiration using a cutter under endoscopic observation (Fig. 3b). OQrimo^®^ maintained stable endoscope positioning in this case, enabling observation of the peripheral retina without scleral indentation. No complications occurred during the intraoperative or postoperative periods. The postoperative recovery was favorable, and despite the preoperative emulsification of silicone oil, there was virtually no residual oil.

### Case 8

The third patient, a 15-year-old woman, was diagnosed with monoarticular juvenile idiopathic arthritis at the age of two. She developed uveitis when she was three and began treatment with methotrexate and folic acid. At the age of eight, she underwent bilateral cataract surgery, and adalimumab was introduced in 2020; however, the inflammation remained poorly controlled. At age 13, her right intraocular pressure (IOP) rose to 25 mmHg, leading to a diagnosis of secondary glaucoma. That same year, trabeculectomy was performed on her right eye, but insufficient IOP control made the decision to proceed with Ahmed valve implantation via pars plana. The preoperative right IOP measured 26 mmHg and showed a glaucomatous visual field defect.

**Fig. 4:**
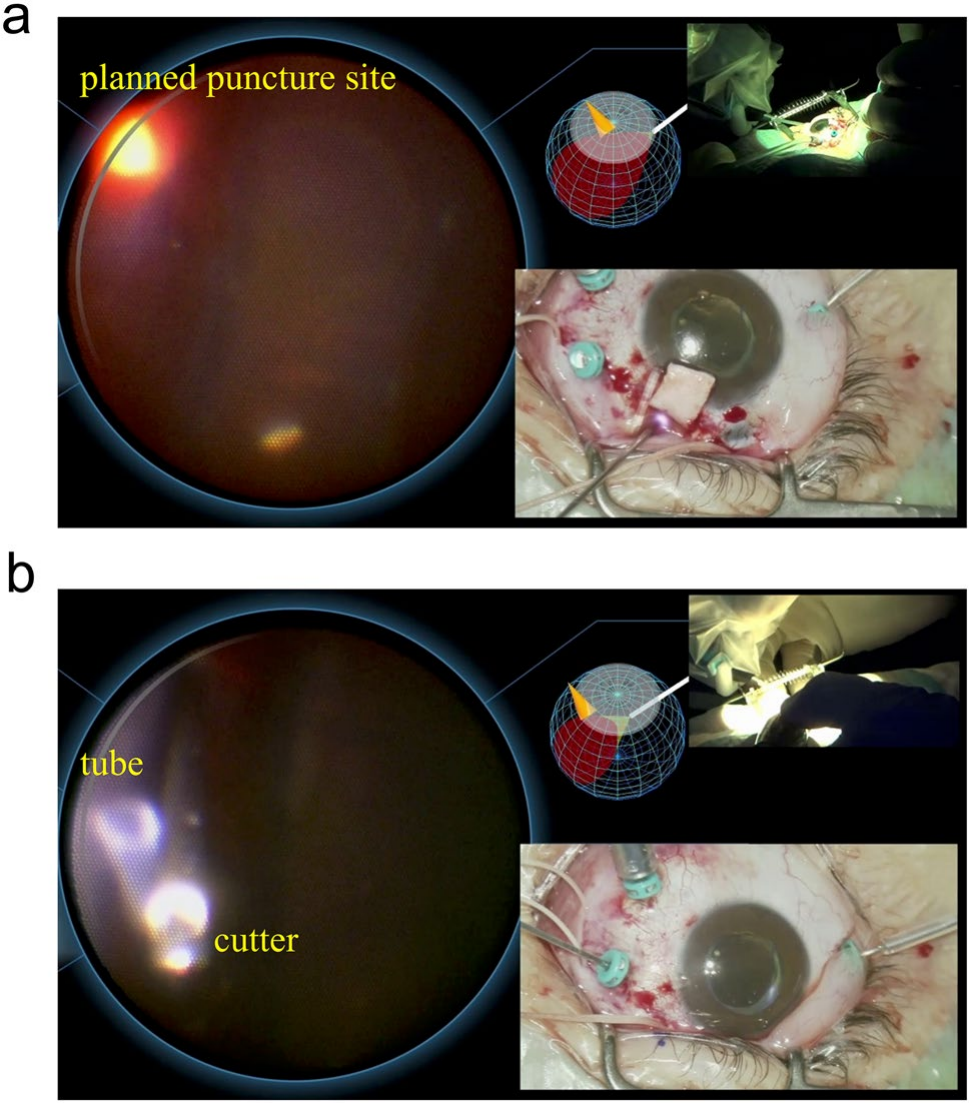
Use of OQrimo^®^ in Case 8.** a** Intraocular illumination was applied through the sclera at the planned puncture site (lower right panel). We could confirm that the planned puncture site was within the pars plana under the endoscope (left panel). Each device is used without interference (upper right panel). **b** The vitreous body around the puncture site was adequately dissected under the endoscope (left panel). No scleral indentation is being applied (lower right panel). Each device is used without interference (upper right panel).

PPV was performed under general anesthesia, four 25G ports were created, and PPV was conducted. A 3x6 mm scleral flap was made 6 mm from the nasal superior corneal limbs, and an Ahmed valve was positioned 9 mm from the limbs. The irrigation port was repositioned from the inferior temporal side to the inferior nasal side. The endoscope was held by the OQrimo^®^ and inserted through the inferior temporal port. Intraocular illumination was applied through the sclera at the planned puncture site (Fig. 4a). We confirmed that the planned puncture site was within the pars plana under the endoscope (Fig. 4a). The sclera was then punctured with a 23G needle. The tube was inserted into the eye, with the vitreous surrounding the tube excised under endoscopic guidance, and the scleral flap was sutured. The vitreous body and proliferating membrane at the puncture site were adequately dissected under the endoscope without scleral indentation (Fig. 4b). No complications arose intraoperatively or postoperatively. The postoperative course was satisfactory, and at one month postoperatively, the right IOP remained in the low teens.

## Discussion

This study reports the case characteristics and features of OQrimo^®^ in its initial clinical use. In intraocular endoscopic applications, OQrimo^®^ was found to be safe for the human eye and could be used without significant interference with the RESIGHT^®^ wide-viewing system. None of the eight patients included in this study experienced intraoperative or postoperative complications. To date, no long-term complications have been reported in ophthalmic robotic surgery, and our eight cases using OQrimo^®^ [9] demonstrate its efficacy.

OQrimo^®^ is designed for use in combination with RESIGHT^®^, a wide-angle fundus viewing system, and was successfully implemented in our case series. While RESIGHT^®^ provides a bird’s-eye view of the ocular fundus, it does not allow for peripheral visualization. The endoscopic mode of OQrimo^®^ compensates for this limitation by enhancing the wide-viewing system [10, 11].

Kita et al. highlight the advantages of an endoscopic approach, which enables observation of the peripheral retina, vitreous base, and pars plana without excessive ocular manipulation or pressure [12]. Techniques such as endoscopic vitrectomy and endoscopic ciliary photocoagulation have also been developed, suggesting that endoscopic vitreoretinal surgery may be beneficial [13–16]. In this case series, the peripheral retina, ciliary body, posterior surface of the iris, and intraocular lens were observed without indentation using only OQrimo^®^, fully leveraging the advantages of the endoscopic system.

Although not applied this time, OQrimo^®^ can also be used simultaneously with a 3D heads-up visualization system. The simultaneous display of endoscopic and wide-field 3D images on a monitor is reported to enhance ergonomics, maintain probe orientation, and assist in guiding surgical movements with the endoscope [17, 18]. Additionally, 3D visualization systems have been shown to provide comparable surgical outcomes and superior contrast ratios in vitreoretinal surgery compared to traditional eyepiece microscopes [19, 20]. These findings suggest that combining a 3D visualization system with OQrimo^®^ may enhance surgical precision and overall quality.

Although the endoscopic mode of OQrimo^®^ was used in the present case series, the application of the light-guide mode and its combined use with a 3D visualization system warrant further investigation. In particular, the potential of these new modes to revolutionize surgical techniques should be evaluated through additional clinical studies.

**Fig. 5:**
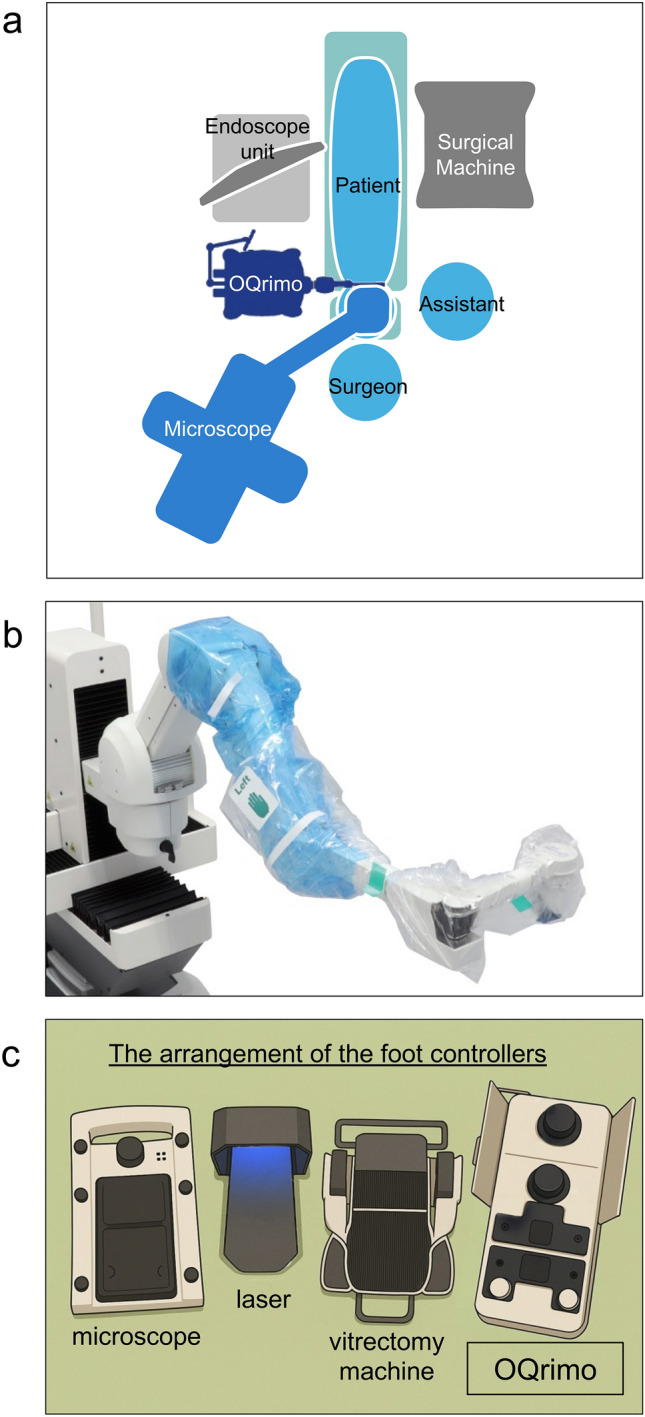
In the operation room.** a** Equipment layout in the operating room. The OQrimo^®^ can be placed on either side of the patient's head, but an assistant should be positioned on the opposite side. The microscope should be placed behind the surgeon. The endoscope unit should also be placed behind the OQrimo^®^ to allow smooth viewing of the endoscopic images. **b** Draping feature of OQrimo^®^. **c** The arrangement of the foot controllers: from left to right, the pedals for the microscope, laser, vitrectomy machine, and OQrimo^®^ are positioned in sequence.

The currently known limitations of OQrimo^®^ include the need for careful operating room arrangements (Fig. 5a), a time-consuming preoperative setup (e.g., transporting the device into the operating room, connecting plugs, setting up the display, and draping (Fig. 5b)), complex foot controller operation, and potential misalignment between the patient’s face and the endoscope. OQrimo^®^ may require adjustments to the operating room layout. While it can be positioned on either side of the patient’s head, additional space is needed for the vitrectomy machine, endoscope, monitor, and assisting personnel. We arranged the equipment and personnel as shown in Fig. 1c. Moreover, the simultaneous use of multiple foot controllers (for the microscope, surgical machine, laser, and OQrimo^®^) (Fig. 5c) may impact surgical workflow and require a training period for the surgeon to adapt effectively.

Currently, we do not consider the cost-effectiveness of OQrimo^®^ to be high. However, OQrimo^®^ can also be used in procedures like endoscopic cyclophotocoagulation, which has recently become covered by insurance in Japan, opening the possibility for wider adoption. If future technological advances, such as improved resolution of intraocular endoscopes, occur, the use of endoscopes might increase, with potentially boosting demand for OQrimo^®^. Additionally, since OQrimo^®^ can support various intraocular devices beyond endoscopes, it is possible that, in the future, more people will use OQrimo^®^, which could lead to lower prices or expanded insurance coverage, ultimately enhancing its cost-effectiveness.

OQrimo^®^ is a new device that has been proven safe for use. Although it requires a certain level of familiarity with its different operating procedures, we will continue to utilize and evaluate OQrimo^®^, reporting new findings as it holds promise for developing innovative techniques and enhancing existing procedures.
